# The Effect of Gum Arabic Supplementation on Growth Performance, Blood Indicators, Immune Response, Cecal Microbiota, and the Duodenal Morphology of Broiler Chickens

**DOI:** 10.3390/ani12202809

**Published:** 2022-10-17

**Authors:** Hani H. Al-Baadani, Rashed A. Alhotan, Abdulaziz A. Al-Abdullatif, Ibrahim A. Alhidary, Abdulrahman S. Alharthi, Saud I. Al-Mufarrej, Maged A. Al-Garadi, Mohammed M. Qaid, Ahmed A. Al-Sagan, Khalid E. Ibrahim, Mahmoud M. Azzam

**Affiliations:** 1 Department of Animal Production, College of Food and Agriculture Science, King Saud University, P.O. Box 2460, Riyadh 11451, Saudi Arabia; 2 King Abdul-Aziz City for Science and Technology, Riyadh 11451, Saudi Arabia; 3 Department of Zoology, College of Science, King Saud University, P.O. Box 2460, Riyadh 11451, Saudi Arabia; 4 Poultry Production Department, Faculty of Agriculture, Mansoura University, Mansoura 35516, Egypt

**Keywords:** broiler chickens, *Acacia Senegal*, performance, gene expression, microbiota, morphology

## Abstract

**Simple Summary:**

Gum Arabic (GA) is considered a natural prebiotic because it contains soluble and indigestible fibers that can stimulate the growth and activity of commensal bacteria through fermentation. Administration of GA in broiler diets may promote early growth as well as the development and health of the intestine and immune functions. However, studies on the mechanism of GA on broiler chickens to improve growth performance and intestinal health are scarce. This study aimed to evaluate the growth performance, internal organs, immune-related gene expression, microbiota, and histological changes of broiler chickens fed with increasing levels of GA. We suggest that GA (0.25 to 0.75%) positively affects the performance, microbiota, immune response, morphology, and gut health of post-hatched chickens.

**Abstract:**

Gum Arabic (GA) belongs to the Fabaceae family and contains indigestible soluble fibers (80–85%) that could be fermented by commensal bacteria to enhance performance, immune response, and intestinal integrity. This study aimed to investigate the effects of GA on performance, serum biochemical indicators, microbiota, immune-related gene expression, and histological changes in chickens. Six GA levels (0.0, 0.12, 0.25, 0.5, 0.75, 1.0%) were allocated using a total of 432 1-day-old male chickens (12 replicates with 6 chickens each). Growth performance was evaluated on days 10 and 24 of age. Blood parameters, organ pH levels, and intestinal health were determined on day 10 of age. Results showed that GA at 0.12% increased weight gain and 0.12 to 1.0% decreased feed intake but was best in feed conversion ratio and production efficiency except for 1.0% on day 1–10 of age. There was an increase in the thymus weight at GA level 0.25 to 0.75%. GA decreased the pH value of the proventriculus (at 0.50 and 1.0%) as well as the duodenum and cecum (at 0.12 and 1.0%). Chickens fed GA between 0.25 to 1.0% had higher protein and HDL, but lower cholesterol, LDL, and creatinine. Globulin was increased at 0.50% GA, while glucose and triglycerides were decreased (at 0.25 and 0.75% GA, respectively). The immune-related gene expression was reduced, except for 0.25% GA, which increased IL-10. Furthermore, chickens fed GA (0.25 to 0.75%) had higher *Lactobacillus* spp. and lower *Salmonella* spp. and *Escherichia coli*. When chickens received GA, the villus length and length to crypt ratio were higher, which also improved the integrity of intestinal epithelial cells and early duodenal development. We conclude that using GA (0.25 to 0.75%) as a natural prebiotic positively affects the performance, microbiota, immune response, morphology, and gut health of post-hatched chickens. More studies are needed to determine the potential mechanism of GA on broiler chickens.

## 1. Introduction

The inclusion of antibiotic growth promoters (AGP) in poultry feed has long been practiced worldwide due to their capacity to enhance intestinal health, prevent pathogenic bacteria and promote growth [1]. However, the use of AGP has declined substantially due to bacterial resistance, an imbalance in microflora, and increased consumer demand for poultry products free of antibiotics [2]. Schokker et al. [3] reported that AGP application in the first days post-hatching affects microbial colonization negatively in broilers. In response, the European Union banned the use of AGP in poultry feed [4]. All of these concerns prompted researchers to look for the safest and most natural diet supplements, such as prebiotics, medicinal plants, and herbal products [5,6]. On the other hand, dietary supplements could be a useful strategy for promoting early growth and gastrointestinal development in broiler chickens [7].

Gum Arabic or GA (*Acacia Senegal*) is a natural supplement obtained from the exudate of tubers or tears of Acacia species [8]. The U.S. Food and Drug Administration has recognized GA as one of the safest dietary fibers for humans [9]. GA is considered to be a natural prebiotic as a result of containing indigestible fibers such as polysaccharides, neutral sugars (rhamnose, arabinose, and galactose), and glucuronic acid that selectively stimulate the growth and activity of beneficial bacteria by fermentation of the caecum besides containing organic matter, amino acids, and minerals [10]. Previous research has shown that GA at levels up to 6% as a broiler feed ingredient effectively acts as a prebiotic in improving growth performance and gut health [11]. Dietary supplementation with GA (0.1 to 2%) improved biochemical serum indicators in rabbits [12] and rats [13]. Sharma et al. [14] reported that GA at 0.25 to 2% has antimicrobial activity. However, increasing beneficial bacteria and eliminating pathogens by GA leads to a healthy gut, which may appear in the growth performance of broiler chickens [15]. A study by Teng and Kim [16] indicated that GA as a natural prebiotic lead to improved gut health by promoting lactobacilli in young chickens. Moreover, using GA in the chicken diet after hatching (1 to 10 days old) successfully enhances the gastrointestinal tract’s early growth and development. [17]. 

To our knowledge, published reports on the efficacy of GA supplements in broiler chickens are scarce, and their effects on the microbiota, development, and health of the chicken intestine have not been elucidated. The present study hypothesized that dietary supplementation with GA as a natural prebiotic could have beneficial effects on gut health by activating beneficial bacteria and promoting early gastrointestinal tract development, which could be reflected in improved growth performance of broiler chickens. Therefore, this study aimed to investigate the effects of GA on growth performance, weight of internal organs, pH levels of gut segments, immune-related gene expression by real-time quantitative PCR, counting microbiota colonies of cecal, and duodenal morphology of broiler chickens.

## 2. Materials and Methods

The King Saud University’s Scientific Research Ethics Committee (SREC) gave its approval to the current study and the use of all chickens (Ethics reference number: KSU-SE-20-39).

### 2.1. Chemical Composition Analysis

Gum Arabic or GA (*Acacia Senegal*) was purchased as natural product from local company (Abnaa Sayed Elobied Agro Export, Khartoum State, Sudan). It was ground into a fine powder at the College of Food and Agriculture Sciences, King Saud University, Saudi Arabia. Nutrient composition analysis of GA powder and feed samples (starter and grower) was carried out according to the methods of AOAC International [18]. Amino acid content was analyzed using high-performance liquid chromatography (HPLC) according to the method described previously [19]. All minerals were determined by an atomic absorption spectrometer system (PerkinElmer, instruments, AAnalyst, Shelton, USA). Sugar derivatives of GA were analyzed by the procedures described by Gashua et al. [20] and Elsayed et al. [21] using gas chromatography-mass spectrometry (GC-MS; Agilent Technologies, Palo Alto, CA, USA). Bioactive compounds were expressed as a percentage of extracted GA.

### 2.2. Housing Chickens and Experimental Design

A total of 432 one-day-old male broiler chickens (Ross 308) purchased from a hatchery near Riyadh City were used in this study. The chickens were randomly divided into 6 GA groups (basal diet was supplemented with 0.0, 0.12, 0.25, 0.5, 0.75, 1.0% GA) with 12 replicates (6 chickens per replicate). The basal diet was formulated in the mash form at two stages: starter (1–10 days) and grower (11–24 days), according to the nutrient requirements of the Ross 308 Management Guide recommendations (Aviagen, 2019, New York, NY, USA). The feed ingredients and nutrient composition of the basal diets are listed in Table 1. Feed and water were provided ad libitum to the chickens until the end of the study period. All chickens were reared in environmentally controlled battery cages where the temperature and humidity were 35 °C and 65% on the first day and then gradually decreased (2 °C per 3 days) until they reached 24 °C and 50% after 24 days, according to standard management practices. The lighting program was continuous for 24 h during the first week after which 23 h of light and 1 h of darkness were maintained throughout the study. All chickens were vaccinated against NDV, IBV, and IBDV (Fort Dodge Animal Health-USA).

### 2.3. Performance Measurements 

At one day of age, chickens were weighed individually to obtain an equal initial body weight per replicate in each level (group). Chickens and feed were weighed at the end of the nutritional stages after 10 days (starters) and after 24 days (growers) to calculate body weight gain, total feed intake, and feed conversion ratio [22]. In addition, the European index for production efficiency was evaluated as (the sum of live weight (%) multiplied by the live weight (kg), then divided by age in days multiplied by feed conversion ratio (*g*/*g*)) × 100 according to Goiri et al. [23].

### 2.4. Relative Weight of Some Internal Organs

At 10 days of age, the internal organs, such as proventriculus, gizzard, thymus, bursa, spleen, liver, heart, pancreas, kidney, and small intestine, were removed from 12 chickens of each treatment group selected for slaughter and weighted separately. The relative weights of the different organs were calculated as a percentage based on live weight [24]. 

### 2.5. Determine the pH Values of Various Gastrointestinal Tract Segments

The pH values of the segments of the gastrointestinal tract (proventriculus, gizzard, duodenum, jejunum, ileum, and cecum) were determined directly in the lumen without touching the walls during the slaughter period of the chicken in duplicate using a method previously described by Zanu et al. [25]. The pH was measured using a microprocessor-controlled digital pH meter (model pH 211; Hanna Instruments, Woonsocket, RI, USA).

### 2.6. Serum Biochemical Analysis

Blood samples from 12 chickens per treatment group were collected in tubes without EDTA at 10 days of age. Serum was separated by centrifugation at 3000× *g* for 15 min for biochemical analysis. Total protein, albumin, glucose, total cholesterol, high-density lipoprotein (HDL), triglycerides, and creatinine were determined spectrophotometrically (Randox, London, U.K.) using reagent kits (Randox, London, UK) according to the manufacturer’s instructions. Serum globulin concentration was determined by subtracting albumin concentration from total protein [26]. To determine low-density lipoprotein (LDL), the following formula was used: LDL = triglycerides – HDL − (triglycerides/5), according to Panda et al. [27].

### 2.7. Immune Response 

Total mRNA was extracted from 10 samples (proximal upper jejunum) per treatment group according to the protocol of the ZymoQuick mRNA kit (Quick-RNA Miniprep, CA, USA). The quantity and quality of mRNA extracted were measured by a Nanodrop spectrophotometer (Thermo Scientific, 2000 Nanodrop, Waltham, MA, USA). Synthesis of complementary DNA (cDNA) was performed using the cDNA Reverse Transcription Kit (Applied Biosystems, Thermo Fisher Scientific, Foster, CA, USA). Gene expression of cDNA was determined using a Real-time quantitative PCR system (7300 Real-Time PCR system, Applied Biosystems) with primer sequence of target genes: IL-4 (F: GCCACCATGAGAAGGACACT and R: ACTCTGGTTGGCTTCCTTCA), IL-6 (F: CTGCTCCTCGTGATGGCTAC and R: CCGAGGATGTACTTAATGTGCTG) and IL-10 (F: GACGTAATGCCGAAGGCAGA and R: TGCTCTTGTTTTCACAGGGC) according to the Basic Local Alignment Search Tool. Each reaction was performed in duplicate for each target gene with added SYBR Green PCR Master Mix (Applied Biosystems, Thermo Fisher Scientific, Foster, CA, USA) and calculated for fold change in gene expression using the 2^−ΔΔCt^ method [28].

### 2.8. Cecal Microbiota

Cecal digesta samples were collected from 10 chickens per treatment group in a sterile 1.5-mL Eppendorf tube and stored (−20 °C) until analysis to count microbiota colonies according to the method of Azzam et al. [6]. Approximately one gram of the cecal content was serially diluted in 9 mL of buffered peptone water (1:10) until the desired dilution was achieved. The colonies were clear and easy to count (50 to 300 colonies). From each dilution, 0.1 mL was cultured on selective media for the bacterial species studied. Selective agar media were used for the enumeration of bacterial target groups such as *Lactobacillus* spp. on de Man, Rogosa, and Sharpe agar (MRS, Himedia, Mumbai, India), *Clostridium perfringens* on Brain Heart Infusion (BHI, Oxoid, Milan, Italy) agar, and anaerobic bacteria by plate counting agar (incubated at 37 °C with 5% CO_2_ for 24 h), while total aerobic bacteria by plate counting agar (Himedia, Mumbai, India), *Salmonella* spp. and *Escherichia coli* by eosin methylene blue agar (EMB, Hardy Diagnostics, Santa Maria, CA, USA) to distinguish the two microbes (incubated at 37 °C for 24 h) according to the method of Qaid et al. [29]. Colonies were counted using a colony counter, and results were expressed as log^10^ colony forming units per gram. 

### 2.9. Duodenum Histomorphometry and Histopathological

Histomorphometric of the duodenum were prepared according to the method described by Daneshmand et al. [30]. A sample approximately 2 cm long was taken from the medial part of the duodenum (sample/chicken) to obtain 12 samples for each treatment group. After cutting, the samples were rinsed with sterile normal saline (0.9% NaCl) without stressing the tissue wall and placed in neutral buffered formalin (10%) for 72 h to fix the samples. All samples were washed with distilled water to remove excess fixative and then dehydrated in ascending gradations of ethyl alcohol (70–95%) for 60 min each and finally in two changes of absolute ethyl alcohol (100%) for 60 min each and then in two changes of xylene I and II for 60 min each. The fabrics were then impregnated with melted paraffin wax at 60 °C for 60 min each and embedded in paraffin wax. All previous processing steps were performed automatically using a sample processor (Tissue-Tek VIP 5 Jr, Sakura, Japan). Samples of 5 μm were cut with the Rotary Microtome (Leica Biosystems, RM 2255, Wetzlar, Germany), mounted on slides, and stained with hematoxylin and eosin (Leica, CV5030, Wetzlar, Germany).

Morphometric analysis of villi length and depth of crypts was measured [31] using at least five villi from the sample at 100× using a light microscope (Nikon, Corp., Tokyo, Japan) in conjunction with camera software for image analysis (AmScope digital camera-attached Ceti England microscope, Irvine, CA, USA). The percentage of villus length/crypt depth is calculated [6]. In addition, the histopathological examination in the duodenal of broiler chickens was evaluated using a light microscope according to the method described by Diler et al. [22].

### 2.10. Statistical Analysis

For statistical analysis, the cage mean (6 chickens/cage) was used as the experimental unit for the parameters of growth performance, whereas one chicken per cage was used as the experimental unit for internal organs, pH, serum biochemical profile, cecal microbiota, and histometrics, based on a completely randomized block design. All data were statistically analyzed using one-way ANOVA in SAS software 2008 (Cary, NC, USA) [32]. The Dunnett test (*p* < 0.05) was used to compare GA levels with the basal diet (0.0% GA; control group). It was also examined whether the responses to increasing amounts of GA were linear or quadratic by applying regression analysis. All values were expressed as mean ± standard error of the means (SEM). Pearson A correlation between cecal pH and the microbiota was calculated [33].

## 3. Results

The nutrient content analysis of the basal diet and GA powder (*Acacia Senegal*) are shown in Table 2. GA was rich in gross energy, organic matter, starch, and ash while less in fat. Vital amino acids such as threonine and valine were the most abundant residues. Minerals such as calcium, phosphorus, potassium, magnesium, and iron were the most abundant in GA. In addition, GA extract consists of complex polysaccharides, oligosaccharides, glycoproteins, arabinogalactans, and monosaccharides (Table 3).

The effects of GA on the general growth performance of male broiler chickens from 1 to 24 days of age are shown in Table 4. According to Dunnett’s test, the results show that adding GA at 0.12% of the basal diet had the highest weight gain during the starter phase (*p* = 0.004) compared with the basal diet (0.0% of GA). The feed intake and feed conversion ratio were reduced by 0.12, 0.25, 0.75, and 1.0% of GA (p = 0.001) except for 1.0% GA which did not affect feed conversion ratio compared with the basal diet. Additionally, there was an observed quadratic response (*p* = 0.007) of GA on weight gain and linear response on feed intake, feed conversion ratio, and production efficiency index upon increasing GA levels (*p* = 0.004, *p* = 0.001, *p* = 0.007, respectively). For the grower phase (11 to 24 days of age), the 0.12 and 0.25% GA groups consumed less feed, gained more weight and had a reduced feed conversion ratio compared with the control group. Feed intake and weight gain of the chickens responded in a quadratic fashion to increasing GA levels. In contrast, the feed conversion ratio responded linearly with increasing GA levels (*p* = 0.001). The production efficiency index was increased for the groups that received 0.12 up to 0.50% of GA compared to the control group during the starter and grower phases. During the overall period (1 to 24 days of age), both 0.12 and 0.25% of GA resulted in lower feed intake and feed conversion ratio and higher production efficiency index (for 0.12% inclusion only) compared with chickens fed a basal diet (*p* < 0.05). On the contrary, weight gain was not influenced nor showed any linear or quadratic response to GA levels at 1 to 24 days (*p* > 0.05).

The effects of GA on the internal organ relative weight and pH values of the gastrointestinal tract segments are shown in Table 5. The relative weights of all internal organs were not influenced nor showed any linear or quadratic responses by GA levels (*p* > 0.05) except for the relative weight of the thymus, which was increased for the chickens that received GA at 0.25 to 0.75% compared to the control. Thymus weight also increased (*p* = 0.002) gradually in a quadratic way to reach a maximum response at 0.75% GA, then dropped thereafter. The pH values of gizzard, jejunum, and ileum segments were not influenced (*p* > 0.05) nor did they show any linear or quadratic responses when GA levels were increased in the diets. On the contrary, pH was lowered when chickens received GA at 0.50 and 1.0% in the proventriculus, 1.0% in the duodenum, and 0.12 and 1.0% in the cecum compared to the control group. Additionally, there was an observed linear response to GA levels of in the duodenum and cecum (*p* = 0.006, *p* = 0.005; respectively).

The effects of GA on the measurement of blood parameters in male broiler chickens are shown in Table 6, including 0.25% or more of GA increased total serum protein and HDL concentrations (*p* = 0.001, *p* = 0.002; respectively) but lowered total cholesterol and LDL (*p* = 0.001) compared to the basal diet. The 0.50% GA group was associated with a higher serum globulin concentration compared to the control. The groups 0.25% and 0.75% GA were found to have lower glucose and triglyceride concentrations, respectively (*p* < 0.05). Serum creatinine concentrations decreased (*p* = 0.001) when chickens received 0.12 or more GA compared to the control. Additionally, there was a quadratic response in total protein, globulin, glucose, total cholesterol, LDL, triglycerides, and creatinine concentrations with increasing GA levels. Positive linear relationships in HDL and albumin concentrations were observed due to GA supplementation. 

**Figure 1:**
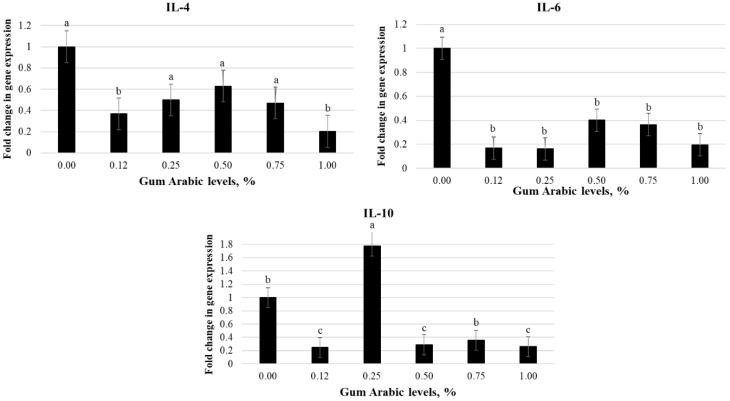
Effect of gum Arabic (GA) on immune-related gene expression of male broiler chickens on day 10 of age. IL-4 = Interleukin 4 (*p*-value: GA = 0.020; L = 0.002; Q = 0.662). IL-6 = Interleukin 6 (*p*-value: GA = 0.001; L = 0.001; Q = 0.008). IL-10 = Interleukin 10 (*p*-value: GA = 0.001; L = 0.051; Q = 0.321).

The effects of GA on immune-related gene expression of male broiler chickens are shown in Figure 1. The 0.12 and 1.0% groups had a lower fold change in IL-4 expression than the control group (*p* = 0.020) and an observed negative linear response with levels of GA (*p* = 0.002). The fold change in the expression of IL-6 was reduced when chickens received GA compared to their counterparts in the control group (*p* = 0.001), with a quadratic response observed (*p* = 0.008). Furthermore, the expression of IL-10 was reduced in chickens that received 0.12, 0.50, and 1.0% GA and increased in chickens that received 0.25% GA compared to the control (*p* = 0.001) but did not show any linear or quadratic responses to GA levels (*p* > 0.05).

Aerobic and anaerobic bacteria and *Clostridium perfringens* were not influenced by GA supplementation (*p* > 0.05), as shown in Table 7. The inclusion of 0.25 or more of GA resulted in a higher *Lactobacillus* spp. count and lower *Salmonella* spp. compared to the control (*p* = 0.001, *p* = 0.009, respectively). Only the 1.0% GA group had a higher *Escherichia coli (p = 0.010),* and the 0.25% GA had a higher *Lactobacillus* to *Escherichia coli* ratio (*p* < 0.030) than the control. In addition, there was an observed linear response of GA levels on *Salmonella* spp. count and a quadratic response on *Lactobacillus* and *Lactobacillus* to *Escherichia coli* ratio upon increasing GA levels (*p* < 0.05). The results of correlations between the cecal microbiota and pH values are shown in Table 8. A strong negative correlation was observed between *Lactobacillus* count and cecal pH values (*p* = 0.010). The other bacterial populations (*Clostridium perfringens, Escherichia coli and Salmonella Typhimurium*) showed no correlations (*p* > 0.05) with the pH values of the cecum.

The effects of GA on the duodenal histometric measurements of male broiler chickens are shown in Table 9. Chickens that received 0.12 to 1.0% GA had higher villus length and villus length to crypt depth ratio compared to the basal diet (*p* = 0.001, *p* = 0.001, respectively). Only the 0.25% group had a higher crypt depth than the control group (*p* = 0.001). Additionally, there was an observed quadratic response of GA levels to villus length, crypt depth, and villus length to crypt depth ratio upon increasing GA levels (*p* < 0.05).

**Figure 2:**
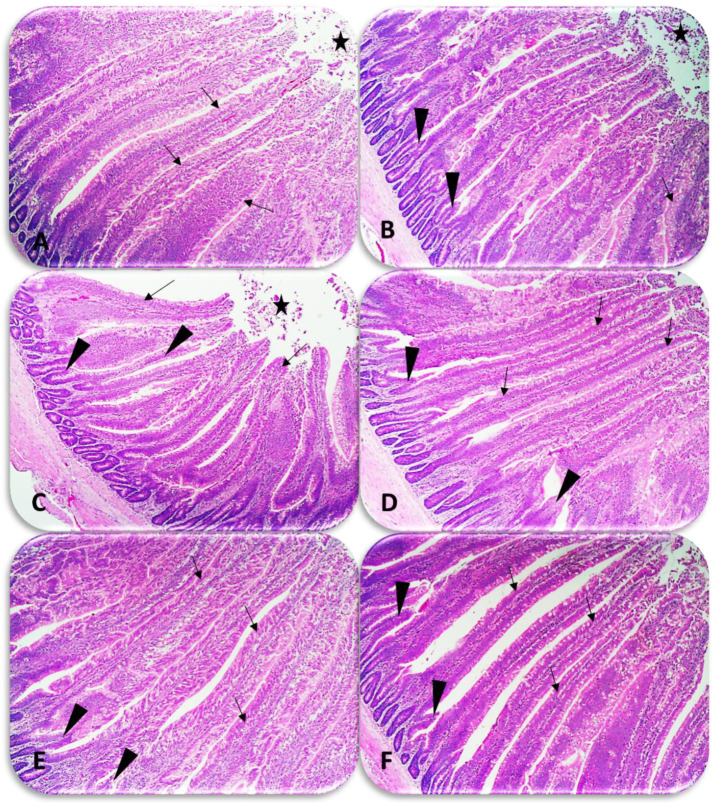
Representative photomicrographs of histopathological examination of broiler chicken’s duodenum samples on d 10 of age stained with hematoxylin and eosin (100×). (**A**): duodenum of chickens (0.00% GA) showing proliferative and some denuded villus enterocytes with abundant goblet cells (arrow), desquamated epithelial sheets inside the lumen (star). (**B**): 0.12% GA, villus structure was nearly high with abundant goblet cells, still found desquamated epithelial sheets in the lumen (star), and proliferative enterocytes (arrow) with crypts appeared to have high compensatory regenerative activity (arrowhead). (**C**): 0.25% GA showing the enterocytes and all villus coats were nearly better than 0.0 and 0.12% GA (arrow) with still faint mucus and desquamate in the lumen (star). Clear regeneration events from deeper intestinal crypts (arrowhead) was observed. (**D**–**F**): levels 0.50, 0.75, and 1.0% GA, respectively, morphological appearance, including villus coats and enterocytes layers were improved than chickens of other GA levels (arrow) and all crypts have regenerative activity (arrowhead).

The effects of GA on the duodenal histopathological examination of broiler chickens are shown in Figure 2A–F. In chickens fed the basic diet (A), proliferative enterocytes are seen raised besides the goblet cells, which also include hyperplastic association and activated crypts with mild thickening of some villi. In addition, a mucinous exudate mixed with desquamated epithelial sheets was noticed in the intestinal lumen within samples. For the chickens that received 0.12% GA (B), the villi structure was improved compared with the control chickens, which characterized by many criteria including thickening of villi was seen and desquamated epithelial sheets inside the intestinal lumen also seen in some samples. The intestinal crypts showed hyperplastic due to increase regenerative. At 0.25% GA (C), most of the villi retained their morphological structure, except still a little mucus seen in the lumen without desquamated sheets. The intestinal villi were shorter and lined by only one layer of enterocytes than the control chickens. Regeneration villi due to more active deeper intestinal crypts were noticed with a fusion of some villi. The duodenum villi structures showed length and thickness in chicken groups receiving 0.50, 0.75, and 1.0% GA (D–F) were consistent with morphological appearance. All duodenal crypts exhibited numerous mitotic activations (marked regenerative index) compared with the basal diet. Local and slight changes in some villi maintained their height and width, but they still atrophied with little separation of villous sheets into the lumen in chickens fed GA at level of 0.50% (D).

## 4. Discussion

Previous studies have confirmed that GA (*Acacia Senegal*) is rich in soluble fiber (galactose, rhamnose, and arabinose), essential amino acids, and minerals [12,34]. Usually, GA especially *Acacia Senegal* is used as a traditional medicine to treat many diseases such as intestinal infections, diabetes, inflammation, and antibacterial agents in humans [35]. The mechanism of action of GA was studied by Kishimoto et al. [36]; Calame et al. [37]; Adil et al. [38]; and Gultermirian et al. [39] in pigs, humans, laying hens, and broilers. They indicated that GA is not degraded in the gastrointestinal tract and thus fermented by the microbiota, which may be reflected in the improved performance and healthy gut of broilers. Our study’s results show adding GA to a basal diet improved overall growth performance, with maximum optimization achieved in chickens fed 0.12% GA during the starter phase. The results agree with those of Tabidi and Ekram [40], who found that chickens receiving GA powder at 0.6% exhibited a higher growth rate and a lower feed conversion ratio. However, cumulative feed intake was lower in the groups fed GA at 0.12 and 0.25%. In contrast, Al-Fadil et al. [41] reported that adding GA at levels up to 6.0% did not affect total feed intake, suggesting that GA acts as a prebiotic when incorporated into the diet and improves the growth performance of broiler chickens. The relative weight of the internal organs of chickens that received GA after 10 days were not affected, except for the thymus gland. This is in contrast to a study by Sato et al. [42], who found that the relative weights of the spleen and bursa were higher in chickens fed GA for 10 days. The pH values were lower in chickens fed GA levels (0.50 and 1.0%) in the proventriculus (0.12 and 1.0%) in the duodenum and cecum compared to the control, which could be due to the physical and chemical properties of GA (relative viscosity = 14.98, pH = 4.69, solubility = 25.66%), resulting in higher acidity in these segments, in addition to its dependence on the amount and buffering capacity of the available feed, which affects the pH range [34].

The biochemical changes in serum are metabolic indicators of health and nutritional status [43]. Total protein (0.25 to 1.0% of GA) and globulin (0.50% of GA) were increased in concentrations compared to the control, which indicates that GA can improve body protein anabolism in chickens. These results agree with Amber et al. [44], who reported that GA at 0.2 to 2.0% of a rabbit’s diet increased serum protein and globulin. The glucose concentration in serum was decreased by GA at a level of 0.25%. This decrease may be attributed to the inhibition of glucose uptake by sodium-glucose transporter 1 in the intestine [45]. The results obtained in this study indicated an improvement in serum lipid profile by GA. Cholesterol and LDL were decreased while HDL was increased at 0.25 to 1.0% GA levels and triglycerides at 0.75% GA compared to the control. In agreement with these findings, Musa et al. [46] reported that GA reduced cholesterol and LDL while increasing HDL levels. This could be due to bile acid absorption disruption in the intestinal (impairing bile acid circulation), resulting in lower serum cholesterol. Another possibility may be that the viscosity increases in duodenum contents with GA levels, which reduces intestinal lipid absorption [47]. The creatinine was decreased with GA levels (0.12 to 1.0%), possibly due to reduced intestinal fluid absorption and enhanced renal functions. Ali et al. [48] indicated that GA was associated with decreased creatinine in healthy mice.

IL-4 and IL-6 function as pre-inflammatory cytokines, while IL-10 is an anti-inflammatory cytokine. This study indicated that GA leads to down-regulation of pro and anti-inflammatory cytokine expressions in chickens through the lowest fold changes in IL-4 expression (at 0.12 and 1.0% GA) and IL-6 expression (at 0.12 to 1.0% GA) and the highest fold change in IL-10 expression (0.25% GA). These results indicate that GA can act as antigens through recognition by immune cell receptors, which beneficially modulate host immunity. Kamal et al. [49] reported that GA increased IL10 and decreased IL-4 and IL-6 in humans. Stabilizing the gut microbiota by increasing beneficial bacteria and eliminating pathogens can promote gut health and modulate host immunity, which may be reflected in broiler chicken growth performance [16]. The microbiota of the cecum is more abundant than the gastrointestinal tract segments and plays a role in the fermentation of indigestible fibers [50]. Teng and Kim [16] indicated that GA improves gut health by promoting *lactobacilli* spp. in young chickens. In our study, chickens that received GA levels (0.25 to 1.0%) had higher *Lactobacillus* spp. and lower *Salmonella* spp. content. Therefore, the pH values of the cecum of chickens were lower than the control. These results agree with the study’s findings that prebiotics increase lactic acid through fermentation and growth of bacterial populations, especially lactobacilli, in the cecum, thereby lowering pH values. Lactic acid is a major byproduct of Lactobacillus bacteria [51]. Our results confirmed this, which showed a strong negative correlation between Lactobacillus count and cecal pH. Pelicano et al. [52] found that the low pH of the lumen inhibited acid-sensitive pathogenic bacteria, such as *Salmonella Typhimurium*, *Clostridium perfringens*, and *Escherichia coli.*

The most commonly used standards to assess nutrient absorption and gut health are villus length, crypt depth, and villus length to crypt ratio [53]. Moreover, broiler chickens have a strong relationship with increased villus height, gut health, and absorption efficiency [54]. High crypt height may indicate increased proliferative activity to compensate for villus height loss [55]. The ratio of villus height to crypt height is a useful measurement for estimating the absorptive capacity of the small intestine, which correlates with increased epithelial cell turnover, and longer villi are associated with activated cell mitosis [56]. In the current study, villus length and villus length to crypt depth ratio were higher in the duodenum, while the crypt depth of villi decreased when chickens were fed GA powder (0.12 to 1.0%) compared to the control group. In agreement with Macari and Maiorka [57], it was shown that the use of fermented prebiotics by bacteria in the cecum increases the height of the duodenal villi of chickens aged 1 to 7 days after hatching. Moreover, the increase in absorbent surface area of villi in the duodenum occurs more rapidly with the age of chickens up to 7 days. The use of GA in chicken diets (1 to 10 days old) is an effective strategy to improve the early growth and development of the gastrointestinal tract. Moreover, the early development of duodenal morphological and functional characteristics of broiler chickens in the initial stage leads to an improvement in early growth performance [17]. GA could be altering the gut microbiota and improving the integrity of intestinal epithelial cells, leading to better absorption of nutrients and hence better growth performance. 

## 5. Conclusions

We concluded that using GA (0.25 to 0.75%) as a natural prebiotic in the diet of post-hatching chickens could be an effective strategy to improve the early growth and development of the gastrointestinal tract by altering the gut microbiota, modulating the immune response, and improving the intestinal epithelial.

## Figures and Tables

**Table 1:** Feed ingredients and nutrient content of the basal diets (%).

Ingredients	Basal Diet	
	Starter (1–10 Day)	Grower (11–24 Day)
Corn	52.66	57.38
Soybean meal 48%	39.10	33.98
Corn oil	3.72	4.41
Dicalcium phosphate	1.82	1.63
Limestone	1.00	0.92
Salt	0.42	0.32
DL-Methionine	0.35	0.32
L-Lysin HCL	0.20	0.19
L-Threonine	0.13	0.11
Premix Blank ^a^	0.50	0.50
Choline CL 60%	0.09	0.09
Sodium bicarbonate	0.01	0.15
Total	100	100
Calculated nutrient, %		
Metabolizable energy, kcal/kg	3000	3100
Crude protein	23.29	21.15
Crude fiber	2.83	2.72
Calcium	0.96	0.87
Non-phytate P	0.48	0.44
Digestible lysine	1.28	1.15
Digestible methionine and cysteine	0.95	0.87
Digestible threonine	0.86	0.77
Digestible arginine	1.43	1.28

^a^ Containing by kg of diets: Vit. A = 2,400,000 IU; Vit. D = 1,000,000 IU; Vit. E = 16,000 IU; Vit. K = 800 mg; Vit. B1 = 600 mg; Vit. B2 = 1600 mg; Vit. B6 = 1000 mg; Vit. B12 = 6 mg; Biotin = 40 mg; Folic Acid = 400 mg; Niacin; 8000 mg; Pantothenic Acid = 3000 mg; Cobalt = 80 mg; Copper = 2000 mg; Iodine = 400 mg; Iron = 1200 mg; Manganese = 18,000 mg; Selenium = 60 mg; Zinc = 14,000 mg.

**Table 2:** Chemical composition analysis of basal diet and gum Arabic (GA) on a dry matter basis ^1^.

Nutrient Analysis, %	Basal Diet		GA
	Starter	Grower	
Gross energy Kcal/kg	3740	3850	4157
Dry matter	92.49	93.23	90.68
Organic matter	86.36	87.27	85.50
Crude Protein	22.21	20.78	2.30
Starch	37.21	37.79	9.32
Crude Fat	7.17	8.70	0.10
Ash	6.13	5.96	5.18
Insoluble fiber	-	-	2.93
Soluble fiber	-	-	80.22
Calcium	1.06	1.15	1.10
Total phosphate	0.79	0.77	0.60
Magnesium	0.21	0.2	0.46
Potassium	1.27	1.21	0.98
Sodium	0.2	0.23	0.02
Iron, ppm	182	205	750
Copper, ppm	21	18	10
Zinc, ppm	97	109	2
Lysine	1.34	1.20	0.06
Methionine	0.68	0.63	-
Threonine	0.92	0.83	0.14
Valine	1.13	1.03	0.13

^1^ The chemical composition analysis was performed in duplicate.

**Table 3:** Analyses of sugar derivatives compounds in gum Arabic (GA) by GC-MS as a percentage of extracted.

RT (min)	Compound Name	% by Area	Mol Weight
20.08	Methyl. alpha. -Arabinofuranoside, 3TMS derivative	10.0	380.1
20.63	D-(-)-Ribofuranose, tetrakis(trimethylsilyl) ether (isomer 2)	12.8	394.2
20.99	D-Ribose, 4TMS derivative	6.4	438.2
21.78	Arabinofuranose, 1,2,3,5-tetrakis-O-(trimethylsilyl)-	9.8	438.2
23.08	Propanoic acid, 2-[4-(1-buten-3-yl) phenyl]-, methyl ester	8.6	218.1
24.49	D-(+)-Talofuranose, pentakis(trimethylsilyl) ether (isomer 2)	6.8	540.2
24.64	D-Arabinopyranose, 4TMS derivative (isomer 2)	38.2	438.2
25.91	Lactose, 8TMS derivative	7.2	918.4

**Table 4:** Effect of gum Arabic (GA) on general growth performance of male broiler chickens from 1 to 24 days of age.

Parameters	GA Levels, %						SEM ^1^	*p*-Value ^2^		
	0.00	0.12	0.25	0.50	0.75	1.00		GA	L	Q
Body weight gain, g										
1–10 d	215 ^b^	231 ^a^	222 ^b^	227 ^b^	220 ^b^	212 ^b^	3.80	0.004	0.122	0.007
11–24 d	889 ^b^	951 ^a^	946 ^a^	945 ^b^	925 ^b^	911 ^b^	15.3	0.040	0.007	0.002
1–24 d	1166	1192	1160	1182	1168	1163.3	18.1	0.866	0.767	0.664
Total feed intake, g										
1–10 d	260 ^a^	245 ^b^	245 ^b^	256 ^a^	249 ^b^	247 ^b^	2.95	0.001	0.004	0.333
11–24 d	1263 ^a^	1176 ^b^	1196 ^b^	1222 ^a^	1233 ^a^	1222 ^a^	14.8	0.003	0.004	0.034
1–24 d	1524 ^a^	1421 ^b^	1442 ^b^	1478 ^a^	1484 ^a^	1469 ^a^	16.2	0.001	0.001	0.033
Feed conversion ratio, g/g										
1–10 d	1.22 ^a^	1.06 ^b^	1.11 ^b^	1.13 ^b^	1.14 ^b^	1.17 ^a^	0.02	0.001	0.001	0.850
11–24 d	1.42 ^a^	1.23 ^b^	1.26 ^b^	1.30 ^b^	1.34 ^b^	1.35 ^b^	0.02	0.001	0.001	0.773
1–24 d	1.31 ^a^	1.19 ^b^	1.24 ^b^	1.25 ^a^	1.27 ^a^	1.26 ^a^	0.01	0.006	0.004	0.678
European Production Efficiency Index										
1–10 d	214 ^b^	260 ^a^	240 ^a^	241 ^a^	233 ^b^	220 ^b^	5.63	0.001	0.007	0.818
11–24 d	341 ^b^	416 ^a^	403 ^a^	396 ^a^	376 ^b^	367 ^b^	10.3	0.001	0.001	0.106
1–24 d	387 ^b^	433 ^a^	404 ^b^	409 ^b^	398 ^b^	398 ^b^	10.0	0.047	0.067	0.911

^a,b^ Means that do not share a common superscripted letter within a row for each parameter differ significantly from those of the basal diet (0.0%), as determined by the Dunnett test (*p* < 0.05). ^1^ SEM = Standard error of means for diet effect. ^2^ GA = gum Arabic response; L = linear response; Q = quadratic response.

**Table 5:** Effect of gum Arabic (GA) powder on the relative weight of internal organs and pH values of gastrointestinal tract segments in male broiler chickens on d 10 of age.

Parameters	GA Levels, %						SEM ^2^	*p*-Value ^3^		
	0.00	0.12	0.25	0.50	0.75	1.00		GA	L	Q
Internal organs ^1^										
Proventriculus	0.87	0.84	0.82	1.04	0.89	0.90	0.05	0.105	0.634	0.696
Gizzard	2.99	2.74	3.06	3.13	2.97	2.82	0.10	0.176	0.678	0.374
Thymus	0.29 ^b^	0.39 ^b^	0.46 ^a^	0.52 ^a^	0.56 ^a^	0.43 ^b^	0.03	0.002	0.001	0.002
Bursa	0.19	0.19	0.18	0.21	0.18	0.20	0.01	0.857	0.738	0.565
Spleen	0.08	0.09	0.09	0.08	0.09	0.07	0.004	0.284	0.392	0.051
Liver	3.23	3.09	3.08	3.42	3.48	3.31	0.09	0.057	0.664	0.734
Heart	0.66	0.69	0.64	0.67	0.71	0.70	0.02	0.587	0.403	0.621
Pancreas	0.52	0.52	0.46	0.48	0.54	0.46	0.02	0.225	0.322	0.710
Kidney	0.72	0.69	0.75	0.80	0.84	0.76	0.05	0.558	0.458	0.662
SmallIntestine	9.6	9.2	8.2	9.2	8.7	8.9	0.33	0.130	0.040	0.040
PH values of gastrointestinal tract segments										
Proventriculus	4.2 ^a^	4.5 ^a^	4.2 ^a^	3.3 ^b^	4.0 ^a^	3.7 ^b^	0.17	0.013	0.230	0.880
Gizzard	2.8	3.1	2.6	2.7	2.9	3.1	0.12	0.061	0.440	0.210
Duodenum	6.5 ^a^	6.3 ^b^	6.6 ^a^	6.3 ^a^	6.3 ^a^	6.2 ^b^	0.05	0.002	0.006	0.590
Jejunum	6.2	6.2	6.3	6.0	6.1	6.2	0.13	0.801	0.610	0.960
Ileum	6.2	6.0	6.4	5.9	6.0	6.4	0.13	0.145	0.680	0.330
Cecum	6.6 ^a^	6.1 ^b^	6.3 ^a^	6.2 ^a^	6.3 ^a^	6.0 ^b^	0.10	0.007	0.005	0.403

^a,b^ Means that do not share a common superscripted letter within a row for each parameter differ significantly from those of the basal diet (0.0%), as determined by the Dunnett test (*p* < 0.05). ^1^ (g/100 g of live BW). ^2^ SEM = Standard error of means for diet effect. ^3^ GA = gum Arabic response; L = linear response; Q = quadratic response.

**Table 6:** Effect of gum Arabic (GA) on serum biochemical profile of male broiler chickens on d 10 of age.

Parameters	GA Levels, %						SEM ^1^	*p*-Value ^2^		
	0.00	0.12	0.25	0.50	0.75	1.00		GA	L	Q
Total protein, g/dl	3.14 ^b^	3.36 ^b^	3.51 ^a^	3.96 ^a^	3.60 ^a^	3.65 ^a^	0.07	0.001	0.001	0.001
Albumin, g/dl	1.74	1.78	1.94	1.96	2.00	2.12	0.11	0.100	0.038	0.902
Globulin, g/dl	1.39 ^b^	1.57 ^b^	1.56 ^b^	1.99 ^a^	1.60 ^b^	1.53 ^b^	0.10	0.011	0.020	0.015
Albumin/Globulin	1.33	1.15	1.35	1.07	1.40	1.41	0.14	0.626	0.727	0.342
Glucose, mg/dl	253 ^a^	239 ^a^	222 ^b^	239 ^a^	232 ^a^	241 ^a^	6.20	0.050	0.011	0.011
Cholesterol, mg/dl	151 ^a^	158 ^a^	119 ^b^	122 ^b^	99 ^b^	130 ^b^	3.11	0.001	0.001	0.001
HDL, mg/dl	45.3 ^b^	50.0 ^b^	63.7 ^a^	67.6 ^a^	66.9 ^a^	72.7 ^a^	4.14	0.002	0.002	0.234
LDL, mg/dl	73.6 ^a^	77.1 ^a^	24.4 ^b^	25.5 ^b^	7.9 ^b^	24.0 ^b^	3.48	0.001	0.001	0.007
Triglycerides, mg/dl	160 ^a^	155 ^a^	152 ^a^	144 ^a^	118 ^b^	164 ^a^	5.12	0.001	0.026	0.024
Creatinine, mg/dl	0.47 ^a^	0.37 ^b^	0.31 ^b^	0.36 ^b^	0.30 ^b^	0.35 ^b^	0.02	0.001	0.001	0.001

^a,b^ Means that do not share a common superscripted letter within a row for each parameter differ significantly from those of the basal diet (0.0%), as determined by the Dunnett test (*p* < 0.05). ^1^ SEM = Standard error of means for diet effect. ^2^ GA = gum Arabic response; L = linear response; Q = quadratic response.

**Table 7:** Effect of gum Arabic (GA) on cecal microbiota of male broiler chickens on d 10 of age.

Parameters ^1^	GA Levels, %						SEM ^2^	*p*-Value ^3^		
	0.00	0.12	0.25	0.50	0.75	1.00		GA	L	Q
Aerobic	11.4	11.4	11.4	11.5	11.5	11.6	0.25	0.970	0.770	0.790
*Escherichia coli*	7.9 ^b^	8.2 ^b^	7.7 ^b^	8.4 ^b^	8.5 ^b^	8.6 ^a^	0.20	0.010	0.060	0.440
*Salmonella Typh.*	8.9 ^a^	9.0 ^a^	7.9 ^b^	8.0 ^b^	7.9 ^b^	8.0 ^b^	0.27	0.009	0.020	0.110
Anaerobic	10.9	11.1	11.7	12.0	11.9	11.9	0.37	0.180	0.040	0.220
*Clostridium perfringens*	11.1	11.3	10.9	11.5	11.5	11.6	0.32	0.620	0.410	0.760
*Lactobacillus* spp.	9.9 ^c^	10.5 ^b^	11.0 ^a^	11.1 ^a^	11.3 ^a^	11.1 ^a^	0.19	0.001	0.001	0.001
*Lactobacillus*/*Escherichia coli*	1.3 ^b^	1.3 ^b^	1.5 ^a^	1.3 ^b^	1.3 ^b^	1.3 ^b^	0.03	0.030	0.050	0.010

^a,b^ Means that do not share a common superscripted letter within a row for each parameter differ significantly from those of the basal diet (0.0%), as determined by the Dunnett test (*p* < 0.05). ^1^ colonies were counted using a colony counter and the results were expressed as log10 colony forming units per gram. ^2^ SEM = Standard error of means for diet effect. ^3^ GA = gum Arabic response; L = linear response; Q = quadratic response.

**Table 8:** Pearson correlation between cecal pH and bacterial populations.

	Cecal pH Values	
Cecal Microbiota	Correlation Coefficient	
	r_xy_	*p*-Value
*Lactobacillus* spp.	−0.420	0.010
*Clostridium perfringens*	−0.318	0.058
*Escherichia coli*	−0.302	0.073
*Salmonella Typhimurium*	0.125	0.467

**Table 9:** Effect of gum Arabic (GA) on duodenal histomorphometric of male broiler chickens on d 10 of age.

Parameters	GA Levels, %						SEM ^1^	*p*-Value ^2^		
	0.00	0.12	0.25	0.50	0.75	1.00		GA	L	Q
Villus length, μm	833 ^b^	1077 ^a^	904 ^a^	975 ^a^	1054 ^a^	1025 ^a^	16.9	0.001	0.001	0.047
Crypt depth, μm	148 ^b^	159 ^b^	162 ^a^	154 ^b^	157 ^b^	137 ^b^	3.80	0.001	0.161	0.001
Length to crypt depth	8.8 ^b^	13.2 ^a^	14.0 ^a^	10.2 ^a^	12.7 ^a^	12.2 ^a^	0.30	0.001	0.001	0.001

^a,b^ Means that do not share a common superscripted letter within a row for each parameter differ significantly from those of the basal diet (0.0%), as determined by the Dunnett test (*p* < 0.05). ^1^ SEM = Standard error of means for diet effect. ^2^ GA = gum Arabic response; L = linear response; Q = quadratic response.

## Data Availability

The data and analyses presented in this paper are freely available in the thesis of the first author (HHA).
